# The Dental Profession vs. Rubber

**Published:** 1869-04

**Authors:** B. Wood


DENTAL REGISTER.

VOL. XXIII.] APRIL, 1869. [No. 4.

### Original Communications.

### THE DENTAL PROFESSION *vs.* RUBBER.

BY B. WOOD, M. D., D. D. S.

Looking over the late journals we find not only the "rubber question," but the merits of rubber itself as a base for artificial teeth receiving a good share of professional attention. The general expression is adverse to this material for permanent work, and there is manifested a strong inclination to return to metal plates.

We will reproduce the drift of what we find, *pro* and *con.*, preliminary to some remarks on the general question. We will go no further back than the latest numbers of journals before us, and if it should make too lengthy a communication, you, Mr. Editor, may retrench it or place it under the head of "Selections."

In an article on "Mechanical Dentistry," in the *Dental Cosmos* for February, the writer (Dr. J. B. Da Camara, Jr., of Newark, New Jersey) says, speaking of vulcanite or hard rubber: "In point of durability it is unquestionably comparatively worthless. It will rarely wear more than from three to five years intact. After being worn from one to two years, the plate becomes saturated with the fluids of the mouth, the fatty substances contained therein, and those which are constantly coming in contact with it, assisted by the heat of the mouth, decompose the rubber—not to a very great extent, to be sure, but surely sufficient to weaken it materially; it soon gives way, blocks begin to work loose and fall off, with pins intact, having dragged their heads through the rubber. The plate cracks down through the center and eventually breaks in two pieces. Hygienically, I have met many who complain of a heating, drawing sensation, which obtains to such an extent, sometimes, as to produce bronchial affections. Its injurious results upon constitutions peculiarly susceptible to the effects of mercury, which is certainly liberated as the plate undergoes decomposition, and the polution it produces in the very air we breathe by its fetid condition, convince me of its unwholesome nature, and are arguments too conclusive to be refuted. I have never seen a rubber plate that had been worn two years but that was *disgustingly offensive*, and had commenced to show signs of decay.

"Our patrons have been deceived and disappointed by it; they have been cursed by the imposition upon them of charlatans and impostors."

At a late meeting of the "Odontographic Society of Pennsylvania," the merits of rubber underwent a pretty free discussion, as we find in the report of the proceedings.

Dr. Jones said: "The art of manipulating with the hard rubber could be learned in a very few days by almost any one having a slight idea of mechanical work, so that it has now become an injury to the profession by encouraging bunglers." He also objected that the facility with which rubber dentures were obtained by patients had caused them to part prematurely with their natural teeth.

"Dr. W. H. Truman thought rubber to be very rarely as useful as gold or silver. In regard to cleanliness he thought too much had been claimed for vulcanite. In breaking up old cases (a pastime he had indulged in lately) quite a large space is often (indeed, almost invariably) found between the plate and teeth, into which particles of food and the fluids of the mouth find their way. These, in decomposing, give out an odor which, to say the least, is not very pleasant. Sometimes he had found bristles, from the brush used in cleaning the case, worked under the teeth. The rubber being in a measure porous and an organic substance, retains impurities with far greater tenacity than either gold or silver."

Again, "unless made so thick as to be clumsy and awkward in the mouth, there is constant liability to accident. The plates will crack sometimes in spite of every thing, and so will gold or silver, but with the latter the accident can be repaired in a short time, and the case made as strong or stronger than before, while the rubber becomes weaker and more liable to accident every time it is revulcanized, until, after passing through the process three or four times, it becomes entirely worthless.

"As the skill required by the workman in hard rubber is inferior, it had inaugurated slovenly and careless habits; it had enabled men to enter the profession without making the least effort to properly qualify themselves for it.

"Within the past three months he had replaced at least half a dozen vulcanite cases with gold or silver. One gentleman, who has been for many years a practitioner of medicine in this city, for whom a gold set was made in place of one on vulcanite, asserted that he would not have worn the rubber in his mouth for $500, if he had thought, when it was inserted, that his general health would have suffered as it had done since. He now believes it was entirely due to the vulcanite upon which the teeth were mounted. He had worn them a little over three months, and soon recovered his health after discontinuing their use.

"Dr. T. believed that rubber had had its day. The exorbitant, unreasonable and humiliating terms of the Dental

Vulcanite Company are fast driving it into the hands of men without either talent or ability. In their careless hands it will not take long to destroy the reputation that really skillful workmen have made for rubber."

"The President said that the facility with which rubber can be used had lowered the standard of mechanical Dentistry. Again, thousands upon thousands of teeth which might have been preserved have been sacrificed, owing to the limited expense of such dentures in comparison with the cost of saving the natural organs. Again, it is more than probable, owing to the importunities of patients and in opposition to their own convictions, that this material had been too extensively used, even by skillful and experienced workmen. But that it fills a niche, when properly used, quite equal to any other material, must be admitted by all, as, for instance, in cases where there has been great absorption of the alveoli, &c. He did not wish to be understood as advocating its use when it is possible to avoid it, particularly when viewed in its relations to the exactions of the Hard Rubber Company. Regarding gold and silver as materials which have stood the test of time—looking upon Dr. John Allen's continuous gum-work as one of the most valuable inventions of the past twenty years in our profession. Those engaged in mechanical Dentistry will find ample opportunity for the construction of satisfactory operations, and be able to dispense, to a great extent if not entirely, with the use of hard rubber."

"Dr. Long considered that hard rubber had been of very slight, if any, advantage to Dentists. It very rarely seemed to be necessary to use it, as it is seldom equal to gold or silver for a base."

"Dr. Howard favored this material for its ease of manipulation, surety of fit and lightness, where the latter quality is desired or demanded. In durability he did not consider it on a par with gold or silver, and justice to our patients can be rendered with these metals in pretty much all cases."

Dr. Eisenbrey believed, in a great many instances, teeth mounted on rubber can be made to give greater satisfaction than on metals, "and without detriment to the patient's health." He held that "the detriment to the patient wearing a vulcanized plate, was not from the mercury it contains, but owing to the decided want of conducting property of the substance, it being placed among the lowest on the list of nonconducting bodies. A closely fitted plate, together with the drawing of an air chamber, causes an excess of blood to flow to the parts; consequently, an increase of heat is generated, which, not being diffused over all parts of the mouth, the local increase of temperature is retained. Gold and silver being among the best conductors of thermal changes that we have, as fast as there is an excess of heat in the plate, it is conducted over the whole surface of the mouth, thereby keeping it in a state of equilibrium of temperature throughout; hence, there is no uneasy feeling in the hard or soft palate from this cause. The metal plate, he believed, carries off the heat, allows the capillaries to maintain their tenacity and forces the blood on, keeping up in them a normal instead of an abnormal circulation, giving ease and comfort to the patient."

Dr. Breen "likes it better and believes it gives more satisfaction to the patient than gold or the other metals. It has the advantage of being lighter, which reduces the liability to break from falling, and a better fit is procured."

"Dr. Stellwagen desired to be clearly understood that, although in nearly every case he thought that the old-fashioned metal plate was superior to vulcanite, he still recognized that cases might arise when the latter would be preferable under certain co-existing circumstances—then he would recommend the patient to find some one who had the right and ability to make such a piece of work as might be required. He did not use it himself, and thought that the number of instances in which its lauded superiority was urged, were more apt to be due to the readiness with which laziness excuses itself for an imperfect operation, than the result of a truly unbiased comparison of the advantages and disadvantages arising from its use. The testimony of many, some of whom are celebrated for their learning in the medical and other liberal professions, assure us that sometimes a diseased condition supervenes upon the wearing of vulcanite, from some cause or another, which we need not stop to discuss, since it is admitted by even the advocates of the the use of rubber. This fact certainly will, to the conscientious, forbid its employment, except in the very rarest cases, when it seems to be the only thing that can be used. The economy is on the side of the metal plate in at least a ratio of 5 to 2; or, a well constructed set on silver for thirty dollars will, at a moderate estimate, outwear two and a half sets on vulcanite at twenty dollars each."

Dr. C. N. Pierce "had now entirely abandoned the use of rubber, as he was convinced that well made silver sets, heavily galvanized with gold, were far healthier and better in every way than hard rubber, in all but rare cases."

We find, also, in the last DENTAL REGISTER, in a communication by Dr. J. P. H. Brown, of Augusta, Georgia, and in the report of the proceedings of the Ohio State Dental Society, expressions of opinion adverse to rubber upon grounds similar to the above, but as these are already before your readers, we need not reproduce them here.

Now, the Rubber Company come before the profession and demand from fifty dollars to two thousand dollars a year for the privilege of using this material—not, indeed, a definite sum, payable yearly, so much a year, but such as the company see fit to levy each year. It may be fifty dollars this year and two thousand dollars the next year, according as its use may seem to be indispensable to the Dentists, or the extraordinary exigencies of the company may require.

What ought the profession to do in the premises? We incline to the opinion that they ought to *abandon the use of rubber with one accord, and refer their patients who may*

*demand this style of work to skillful and honest rubber-workers, who will not claim the name of "Dentist," nor in any way tamper with the natural teeth.* Some of the reasons in favor of this will be set forth upon another occasion.

